# The need for oral assessment and referral practices tool for palliative patients in Brunei Darussalam: A cross‐sectional study

**DOI:** 10.1002/nop2.591

**Published:** 2020-10-10

**Authors:** Jagjit S. Dhaliwal, Zaidah R. Murang, Hajah A. Haji Husaini, Deeni R. Idris, Munikumar R. Venkatasalu

**Affiliations:** ^1^ Anak Puteri Rashidah Sa'adatul Bolkiah Institute of Health Sciences Universiti Brunei Darussalam Bandar Seri Begawan Brunei Darussalam; ^2^ All India Institute of Medical Sciences Rishikesh India; ^3^ Oxford School of Nursing and Midwifery Oxford Brookes University Oxford UK

**Keywords:** hospice, nurses, nursing care, oral care, palliative, palliative care, terminally ill

## Abstract

**Aim:**

This study aimed to investigate knowledge, experiences, perceptions and barriers of healthcare professionals regarding palliative oral care.

**Methods:**

The study involved 169 palliative care professionals in Brunei. Data collection tool was pretested, validated and self‐administered with sections on demographics; knowledge, attitude and practices; referral of patients; perspectives; and barriers to oral palliative care.

**Results:**

97.3% of participants believed that palliative patients need oral care, and 11.6% of participants were trained in this area. 43.8% were unsure about referral process, and 66.1% of participants had never used a tool to assess oral conditions of palliative patients. Most common oral condition encountered was mucositis (54.5%). 74.1% of participants expected family members to be responsible, and the absence of proper guidelines for assessment (66.1%) was the top challenge in providing oral care for palliative patients.

**Conclusion:**

This study highlights perceptions and experiences of healthcare professionals and need for improved care through development of oral assessment and referral practices tool for palliative patients.

## 1 INTRODUCTION

A high incidence of oral problems has been reported among palliative patients (Ohno et al., 2016). According to published reports, evidence has suggested that the medical management of palliative conditions is likely to produce oral complications among these patients (Saini, Marawar, Shete, Saini, & Mani, 2009). Chemotherapy and the drugs such as bisphosphonates and analgesics have been shown to be associated with oral mucositis and taste disturbances (Davies & Epstein, 2010).

Oral cavity is home for a large number of microorganisms which aggravates the disease process (Mol, 2010). However, healthcare professionals have been shown to hesitate to extend oral care for palliative patients (Soileau & Elster, 2018), or they may not be aware of their responsibilities towards these patients (Mol, 2010). In addition, palliative patients may not complain of discomfort in the oral cavity as they may be physically or cognitively disabled (Fischer, Epstein, Yao, & Wilkie, 2014). These may lead to under‐reporting of oral conditions in palliative patients, which may contribute to failure by healthcare professionals to appreciate the problems.

Despite the significant morbidity caused by oral conditions in palliative patients, research on the perspectives and experiences of healthcare professionals towards palliative oral care is not well‐documented. This study was attempted to investigate the knowledge, experiences, perceptions and barriers of healthcare professionals in Brunei Darussalam setting. It is hoped that the findings from this study can be used in strengthening or redesigning appropriate oral care approaches in enhancing the quality of life for palliative patients.

*Research Question:* To investigate the knowledge, experiences, perceptions and barriers of healthcare professionals in Brunei Darussalam setting.

## 2 MATERIALS AND METHODS

### 2.1 Study design

This cross‐sectional study was conducted from April–June 2019 using the convenient sampling method to recruit all palliative care health professionals which consisted of nurses, doctors and dentists from six health centres in three districts (Brunei‐Muara, Tutong and Belait District) in Brunei Darussalam.

### 2.2 Data collection instrument

This study used paper‐based self‐administered structured questionnaires. As validated questionnaires specific to the research questions could not be found, a new set of questionnaire was designed by the research team based on the qualitative findings of the exploratory study and from the review of the literature. The questionnaires developed consisted of 19 questions and five sections: demographic information; knowledge, attitude and practice of oral palliative care; referral of palliative patients to the dental service for oral care; perspective towards oral palliative care; and barriers to oral palliative care. Each questionnaire required less than 15 min to complete.

### 2.3 Reliability and validity of instruments

The new questionnaires were pretested on 20 health professionals. Their feedbacks regarding any difficulties in answering the questions were noted. The questionnaires were then revised according to the problems identified by using the expertise of the research team which consisted of a dentist (J.S.D.), two palliative care nurses (M.R.V. and A.H.), a clinical nurse (D.R.I.) and a public health researcher (Z.R.), who reviewed the questionnaires according to its relevance, clarity, organization and completeness of the topic.

### 2.4 Participants

Participants were palliative care health professionals which consisted of nurses, doctors and dentists. The eligibility criteria included all health professionals from critical care, geriatrics and palliative care units, and they must be willing to participate.

### 2.5 Sample size

The proposed number of participants was 200 to capture all health professionals working with palliative patients (critical care, geriatrics and palliative care units) in Brunei. However, in the actual study, 169 questionnaires were distributed to six health centres in Brunei.

### 2.6 Research procedures

Permission from the Ministry of Health Research and Ethics Committee (MHREC) and PAPRSB Institute of Health Sciences Research and Ethics Committee (PAPRSB IHSREC) was obtained before starting the study. The purpose, objective and research procedure of the study were explained via briefings to the heads of departments (HoDs) of selected health centres and the involved staffs. A package containing the participant information sheet (PIS), consent form and a set of questionnaire was distributed to the HoDs of selected health centres.

### 2.7 Data analysis

The raw data from the responses of each participant were coded numerically and were entered into IBM SPSS (version 20.0) software for organization, analysis and interpretation. Descriptive statistics were computed for demographic variables, whereas frequency and percentages were calculated for categorical variables, to describe the results for each appropriate research question. Factual presentation of the results included illustrations using tables.

## 3 RESULTS

### 3.1 Demographic characteristics of participants

We recruited 169 health professionals, where 122 responded (response rate of 72.1%) but only 112 were analysed as 10 did not complete the questionnaires. Demographic characteristics of respondents revealed that 64.3% (*N* = 72) of the participants were nurses, 27.7% (*N* = 31) were dentists, and 8.0% (*N* = 9) were doctors.

Most of the participants had diploma (47.3%) as their highest level of educational attainment, followed by bachelors (33.0%), masters (9.8%), doctorate (5.4%) and others (4.5%).

The largest proportion of the participants had 0–5 years of working experience (25.9%), followed by 5–10 years (22.3%), 10–15 years (21.4%), 15–20 years (17.0), and 20 years and above (13.4%).

In terms of workplaces, the majority of the participants worked in Raja Isteri Pengiran Anak Saleha Hospital (RIPASH) (59.8%), 17.9% in The Brunei Cancer Centre (TBCC), 9.8% in National Dental Care (NDC), 5.4% in Suri Seri Begawan Hospital (SSBH), 3.6% in Pengiran Muda Mahkota Pengiran Muda Haji Al‐Muhtadee Billah Hospital (PMMH) and 3.6% in others (Table 1).

**TABLE 1:** Demographic characteristics of study participants

Demographic characteristics	*N*	%
Professional qualification		
Nurse	72	64.3
Dentist	31	27.7
Doctor	9	8
Education		
Diploma	53	47.3
Bachelors	37	33
Masters	11	9.8
Doctorate	6	5.4
Others	5	4.5
Years of experience		
0–5	29	25.9
5–10	25	22.3
10–15	24	21.4
15–20	19	17
20 above	15	13.4
Place of work		
RIPASH	67	59.8
TBCC	20	17.9
NDC	11	9.8
SSBH	6	5.4
PMMH	4	3.6
Others	4	3.6

Note

*N* = number of respondent.

### 3.2 Knowledge, attitude and practices of oral palliative care

This study showed that the majority of participants (97.3%) agreed that palliative patients need oral care, and 78.6% of them had treated palliative patients with oral problems. The majority of participants (48.9%) provided daily oral care for the patients. However, only 11.6% of the participants were trained in managing palliative patients with oral health needs (Table 2).

**TABLE 2:** Responses to knowledge, attitude and practices of oral palliative care questions

Question	Responses								
In your opinion, do you think palliative patients need oral/dental care?	Yes *N* (%)	No *N* (%)	I don't know *N* (%)						
	109(97.3)	0(0)	3(2.7)						
Have you treated palliative patients with oral/dental problems?	Yes *N* (%)	No *N* (%)	Common responses for not treated						
	88(78.6)	24(21.4)	1. Have not encountered a palliative patient 2. Most of the care is given by ward nurses						
How often do you provide oral care for these patients?	Daily *N* (%)	1/week *N* (%)	1/month *N* (%)	Never *N* (%)	Others *N* (%)	Common responses for others			
	48(42.9)	4(3.6)	2(1.8)	24(21.4)	34(30.4)	1. Up to 6 times per day 2. Once every shift 3. Only if referred 4. Seldom			
Have you received any formal training in managing palliative patients with oral health problems?	Yes *N* (%)	No *N* (%)	Responses for training specified						
	13(11.6)	75(67.0)	1. BDS 2. Inter‐ward CNS 3. Oral surgery training						
In your experience, what are the common oral/dental conditions that you have encountered among palliative patients?	Candidiasis	Periodontitis	Caries	Xerostomia	Mucositis	Mouth Ulcers	Cheilitis	Others	Common responses for others
	34	27	59	28	33	61	10	4	1. Retrained roots
	(30.4)	(24.1)	(52.7)	(25)	(29.5)	(54.5)	(8.9)	(3.6)	2. Bleeding
What was/were the most common concern(s) expressed by the palliative patients/caregivers related to conditions of the mouth?	Dry mouth	Food lodgment	Pain	Difficulty swallowing	Dentures problems	Bad breath	Others	Common responses for others	
	60	12	51	33	24	51	9	1. Bleeding 2. Taste change (metallic taste) 3. Infection 4. Ulcers 5 Complication from bactidol	
	(53.6)	(10.7)	(45.5)	(29.5)	(21.4)	(45.5)	(8)		
Have you used any assessment tools/checklist to assess the oral conditions of these patients?	Yes *N* (%)	No *N* (%)	Responses for tools/checklist used						
	14(12.5)	74(66.1)	Caries assessment						
In your opinion, what is the best method to maintain oral hygiene?	Rinsing with saline	Swabbing	Sodium fluoride mouthwash	Teeth brushing	Clotrimazole	Others	Common responses for others		
	18	43	26	66	9	11	1. Bactidol 2. BMX 3. Nystatin 4. Oral 7		
	(16.1)	(38.4)	(23.2)	(58.9)	(8)	(9.8)			
In your opinion, what may be the advantage(s) in administering oral care for palliative patients?	Reduced systemic infection *N* (%)	Increased longevity *N* (%)	Family/patient satisfaction *N* (%)	Better quality of life *N* (%)	Others *N* (%)	Common responses for others *N* (%)			
	71(63.4)	11(9.8)	48(42.9)	54(48.2)	7(6.3)	1. Comfort 2. Pain management			
In your opinion, what may be the disadvantage(s) in administering oral care for palliative patients?	Risk for aspiration *N* (%)	Initiation of infection *N* (%)	Intruding privacy *N* (%)	Others *N* (%)	Common responses for others *N* (%)				
	69(61.6)	12(10.7)	14(12.5)	11(9.8)	1. Bleeding 2. Inconvenience				

Note

*N* = number of respondent.

The top three most common oral conditions encountered by participants were mouth ulcers (54.5%), caries (52.7%) and candidiasis (30.4%). They also reported that dry mouth (53.6%), pain (45.5%) and bad breath (45.5%) as the three most common concerns expressed by palliative patients/caregivers/nurses and doctors related to conditions of the mouth. However, 66.1% of the participants had not used any assessment tools/checklist to assess the oral conditions of palliative patients. Nevertheless, the participants reported that the three best method to maintain oral hygiene in palliative patients were toothbrushing (58.9%), swabbing with chlorhexidine gluconate 0.2%/0.1% mouthwash/gel (38.4%) and using sodium fluoride mouthwash (23.2%) (Table 2).

Most of the participants reported the advantages in administering oral care for palliative patients in reducing systemic infection (63.4%), family/patient satisfaction (42.9%) and better quality of life (48.2%). However, risking for aspiration (61.6%), intruding privacy (12.5%) and risking the initiation of infection (10.7%) were the top three disadvantages in administering oral care for palliative patients as reported by the participants (Table 2).

This study showed that the majority of participants (80.4%) believed that it is important to refer palliative patients to the dental services. When asked about who should be responsible for the oral care of palliative patients, the top three answers given by the participants were family of patient (74.1%), nurse (57.1%) and dentist (50.9%). Participants also reported that clinical findings (such as white or red spots, dryness and bleeding) (71.4%), patients complaints (57.1%) and complaints by family members (44.6%) as the indicators for referring palliative patients to the dental services (Table 3).

**TABLE 3:** Responses to referral of palliative patients to the dental services for oral care questions

Question	Responses						
In your opinion, do you think it is important to refer palliative patients to the dental services?	Yes *N* (%)	No *N* (%)	I don't know *N* (%)				
	90(80.4)	8(7.1)	14(12.5)				
In your opinion, who should be responsible for the oral care of palliative patients?	Dentist *N* (%)	Doctor *N* (%)	Nurse *N* (%)	Family *N* (%)	I don't know *N* (%)	Others *N* (%)	Common responses for others *N* (%)
	57(50.9)	34(30.4)	64(57.1)	83(74.1)	0	10(8.9)	1. Patient 2. Special care dentist
In your opinion, what are the indicators for referring palliative patients to the dental services?	Clinical findings *N* (%)	Complaints by family members *N* (%)	Patients complaints *N* (%)	SOP *N* (%)	Others *N* (%)	Common responses for others *N* (%)	
	80(71.4)	50(44.6)	64(57.1)	44(39.3)	6(5.4)	1. Loose teeth 2. Routine check‐up	

Note

*N* = number of respondent.

### 3.3 Perspective towards oral palliative care

This study showed that the participants were unsure about the referral process (43.8%) of oral care of palliative patients, whereas 30.8% and 28.6% of the participants reported “neglectful” and “not a health priority” as the attitude of the healthcare professionals with regard to the oral care of palliative patients (Table 4).

**TABLE 4:** Responses to perspective towards oral palliative care questions

Question	Responses					
In your opinion, what is the attitude of healthcare professionals regarding the oral care of palliative patients?	Not a health priority *N* (%)	Neglectful *N* (%)	Unsure about referral process *N* (%)	Referral process is complicated *N* (%)	Others *N* (%)	Common responses for others *N* (%)
	32(28.6)	34(30.8)	49(43.8)	17(15.2)	14(12.5)	1. Health priority 2. Lack of knowledge
In your opinion, how do palliative patients respond to the need of oral care?	Lack of compliance *N* (%)	Not a health priority *N* (%)	Neglectful *N* (%)	Lack of knowledge and awareness *N* (%)	Others *N* (%)	Common responses for others *N* (%)
	48(42.9)	44(39.3)	34(30.4)	75(67.0)	5(4.5)	1. Uncooperative 2. Lazy
In your opinion, what is the attitude of caregivers regarding the oral care of palliative patients under their care?	Lack of compliance *N* (%)	Not a health priority *N* (%)	Neglectful	Lack of knowledge and awareness *N* (%)	Others	Common responses for others *N* (%)
	34(30.4)	38(33.9)	32(28.6)	79(70.5)	2(1.8)	1. Health priority

Note

*N* = number of respondent

In addition, participants also reported that both palliative patients and their caregivers were lacking in knowledge and awareness (67.0% and 70.5%) about oral care of the palliative patients. They also tend to not comply (42.9% and 30.4%) and considered oral care as not a health priority (39.3% and 33.9%) (Table 4).

### 3.4 Barriers to oral palliative care

This study showed that the majority of participants (78.6%) reported challenges in providing oral care for palliative patients. The top three challenges reported were the absence of proper guidelines for assessment (66.1%), manpower constraints (57.1%) and the limited accessibility of patients to oral care (56.3%). The participants also believed that the use of checklist/guidelines to assess oral care of palliative patients (79.5%), formal training for healthcare professionals (79.5%), improved facilities (75.9%) and the presence of designated manpower (61.6%) as ways to improve oral care for palliative patients (Table 5).

**TABLE 5:** Responses to barriers to oral palliative care questions

Question	Responses					
In your opinion, are they any challenges involved in providing oral care for palliative patients?	Yes *N* (%)	No *N* (%)	I don't know *N* (%)			
	88(78.6)	14(12.5)	10(8.9)			
In your opinion, what are the challenges involved in providing oral care for palliative patients?	Manpower constraints *N* (%)	Accessibility of patients to oral care *N* (%)	No proper training of healthcare professionals *N* (%)	No proper guidelines for assessment *N* (%)	Others *N* (%)	Common responses for others *N* (%)
	64(57.1)	63(56.3)	60(53.6)	74(66.1)	4(3.6)	1. No proper equipment 2. Patients refused
In your opinion, how can oral care for palliative patients be improved?	Designated manpower *N* (%)	Improved facilities *N* (%)	Formal training for healthcare professionals *N* (%)	Use of guidelines/checklist *N* (%)	Others *N* (%)	Common responses for others *N* (%)
	69(61.6)	69(75.9)	89(79.5)	89(79.5)	4(3.6)	1. Adequate supplies 2. Presence of dental hygienist 3. Patients’ willingness

Note

*N* = number of respondent.

## 4 DISCUSSION

This study showed that the majority of participants (97.3%) agreed that palliative patients need oral care; however, only 11.6% of the participants were trained in managing palliative patients with oral health needs. Similar cross‐sectional studies also found that 75.8% of Indian nurses and 56.2% of nurses in Riyadh did not receive training in oral care of palliative patients (Al Rababah et al., 2018; Pai & Ongole, 2015). An observational survey among dentists in Australia also found that only 42.3% of them felt adequately trained to manage oral care for patients with cancer, although an overwhelming majority of responders (92.9%) indicated that they were interested in continuing education courses on the subject (Frydrych, Slack‐Smith, Park, & Smith, 2012).

This study also found that the most common oral condition encountered by participants was mucositis (mouth ulcers). This finding is in agreement with a review article on current trends in management of oral mucositis in cancer treatment which reported that mucositis is the most common and dreaded toxicities of palliative patients (Shankar et al., 2017). It has also been reported that approximately 40% of palliative patients undergoing chemotherapy for cancer develop complications in the oral cavity, with half of the patients developing severe oral mucositis (Saito et al., 2014). This suggests that mucositis intervention is an essential component of cancer therapy, as pain due to mucositis can make it difficult for patients to ingest food, leading to malnutrition and lowered immunity (Saito et al., 2014).

To ensure universal first‐class palliative care, it has become necessary to formulate guidelines for daily clinical care and management of these patients (Bausewein et al., 2015). However, this study revealed that 66.1% of the participants had not used any assessment tools or checklist to assess the oral conditions of palliative patients. A review on a critical assessment of oral care protocols for patients under radiation therapy in the regional University Hospital Network of Madrid in Spain also found no clear guidelines for the prevention and treatment of oral illnesses in patients with cancer (Lanzós, Herrera, Lanzós, & Sanz, 2015). Although several oral care guidelines have been published in an attempt to assist the healthcare professionals, experts suggested that they may not always be optimal as clinical practice of oral care varies between and within centres due to the difficulty with implementing guidelines into daily practice, lack of consistency between various guidelines and preference of traditional views over scientific evidence (Elad et al., 2015).

This study also found that the majority of participants (74.1%) had the expectation for family members to be responsible for the oral care of palliative patients. A review article also reported that family members are often expected to assume the role of providing personal care and assisting with symptom management as shorter hospital stays and an increase in outpatient‐provided care seem to have shifted the care of cancer patient to the home (Hazelwood, Koeck, Wallner, Anderson, & Mayer, 2012). However, the same study showed that family members often feel inadequately prepared to provide the care as they are not feeling confident of their knowledge and skills (Hazelwood et al., 2012), a finding similar to the present study where our participants reported that both palliative patients and their caregivers were lacking in knowledge and awareness about oral care of the palliative patients. Another study found that although most of the caregivers asked their care recipients about oral problems infrequently although they reported that their end‐of‐life care recipients’ oral hygiene was their (caregivers) responsibility (Ezenwa et al., 2016). In addition, a study also reported that the attitude of caregivers towards oral health of special needs patients was unsatisfactory and inadequate as most of them thought it was necessary to go for dental check‐up only in case of dental problem (Shah et al., 2017). These findings suggest that future research efforts should focus to improve oral health education and training programmes for caregivers of palliative patients.

Experts had suggested that palliative healthcare professionals should refer patients for a complete oral evaluation if an oral symptom is reported or oral lesion is observed (Elad et al., 2015). However, this study showed that most participants were unsure about the referral process of oral care of palliative patients. Studies on the referral processes of palliative patients for oral care are limited, but a review paper suggested that the related referral pathways are through dental and medical specialists with oncology experience (Samim, Epstein, Zumsteg, Ho, & Barasch, 2016). In addition, a recent study on the perspectives of healthcare professionals also reported barriers to referring patients in an intensive care unit to palliative care (Bluck, Mroz, & Baron‐Lee, 2019). The finding of the present study could be due to the lack of care coordination (Bluck et al., 2019) or the mere absence or unclear guidelines on the referral processes of palliative patients for oral care in Brunei. Therefore, future research should focus on investigating the pathway of referral process for oral care of palliative patients if any, or to formulate an oral palliative care referral practices for palliative patients in order to increase the quality of palliative care in Brunei.

This study also revealed that the absence of proper guidelines for assessment (66.1%) was the top challenge in providing oral care for palliative patients, and they also had suggested the use of checklist/guidelines as the number one way to improve the assessment of oral care for these patients (79.5%). Although several oral care guidelines that have been published in an attempt to assist the healthcare professionals were not optimal due to the reasons stated earlier, a study assessing the effectiveness and of an oral hygiene (OH) protocol in patients with cancer in Italy found that the complications, the risks of infection and permanent oral problems have been minimized in patients undergoing the oral hygiene protocol (Rapone et al., 2016). Riley (2018) also stated that proper assessment of the oral cavity with the implementation of preventative measures should improve oral care of palliative patients (Riley, 2018). These findings showed that oral hygiene protocols can ameliorate and prevent oral complications in patients with cancer, hence improving their quality of life. Therefore, future studies should explore on the common oral conditions among palliative patients in Brunei followed by the development of a tailored assessment tool to guide the healthcare professionals in properly managing oral conditions among these patients.

## 5 CONCLUSION

This paper highlights the need for the development of assessment tools and referral practices package to enhance the quality of care for palliative patients in Brunei. Training of healthcare professionals and educating family members of patients with “Oral palliative care” skills are necessary approaches to empower them to confidently manage oral symptoms of their care recipients.

## CONFLICT OF INTEREST

The authors declare that they have no competing interests.

## AUTHOR CONTRIBUTIONS

ZRM: Draft preparation and data analysis. MRV, HAHH, DRI and JSD: Comments for improvement in writing. All authors read and approved the final manuscript.

## ETHICAL APPROVAL

The study protocol was approved by Ministry of Health Research and Ethics Committee and our institute's research and ethics committee (PAPRSB IHS), based on commonly agreed standards of good practice, such as those outlined in the Declaration of Helsinki Ethical Principles for Medical Research Involving Human Subjects. Participation in the study was through the process of informed consent through the distribution of PIS and written consent form to the participants. The PIS contained the following information: study purpose and goals; procedures and voluntary nature of participation; assurance of confidentiality; the lack of risk for participating; the benefits for participants; and contact numbers for further questions or complaints. In the consent form, the participants were informed that their identities will be kept confidential and will not be revealed in published material and they were free to withdraw from the research project at any time without explanation. Privacy of the respondents was protected by having no identifiers on the returned survey. Questionnaires and other research data will be stored according to the University Research Committee policy and guidelines.

## Data Availability

All data generated or analysed during this study are included in this published article (and its supplementary information files).
